# Epsilon-near-zero nonlinearity enhancement in the extreme ultraviolet

**DOI:** 10.1038/s41377-025-01985-w

**Published:** 2025-10-27

**Authors:** Carino Ferrante, Emiliano Principi, Luca Assogna, Ambaresh Sahoo, Giovanni Batignani, Giuseppe Fumero, Laura Foglia, Riccardo Mincigrucci, Luca Giannessi, Tullio Scopigno, Claudio Masciovecchio, Andrea Marini

**Affiliations:** 1 CNR-SPIN, c/o Dip.to di Scienze Fisiche e Chimiche - Via Vetoio, L’Aquila, Italy; 2 Elettra-Sincrotrone Trieste S.C.p.A., Trieste, Italy; 3 Dipartimento di Scienze Fisiche e Chimiche, Università degli Studi dell’Aquila, L’Aquila, Italy; 4 Dipartimento di Fisica, Università di Roma “La Sapienza”, Roma, Italy; 5 Graphene Labs, Istituto Italiano di Tecnologia, Genova, Italy; 6 Associate, Nanoscale Device Characterization Division, National Institute of Standards and Technology, Gaithersburg, MD USA; 7 Department of Physics and Astronomy, West Virginia University, Morgantown, WV USA; 8 INFN Laboratori Nazionali di Frascati, Frascati (Rome), Italy

**Keywords:** X-rays, Nonlinear optics, Nanophotonics and plasmonics

## Abstract

Materials with a vanishing dielectric constant provide an ideal platform for achieving plasmon-enhanced light-matter interactions and are widely employed in various cutting-edge nonlinear photonics applications. In this study, we present the first experimental demonstration of extreme ultraviolet (XUV) plasmon-enhanced self-driven spectral modification using a submicrometric foil of aluminium. This is achieved through the excitation of widely tunable Ferrell-Berreman epsilon-near-zero resonances with extremely low absorption. Our angle-dependent measurements of spectral modulation enhancement, supported by theoretical analysis, reveal efficient spectral modification at peak intensities as low as 380 GW/cm^2^, which we attribute to ultrafast heating and saturation effects. These findings mark a breakthrough in the enhancement of typically weak nonlinearities in the XUV regime through nonlinear plasmonics, potentially paving the way for unprecedented tools for the manipulation and control of XUV radiation.

## Abstract (web summary)

The first experimental demonstration of extreme ultraviolet (XUV) plasmon-enhanced self-driven spectral modification using a submicrometric foil of aluminium. These findings mark a breakthrough in enhancing XUV nonlinearities.

## Graphical abstract

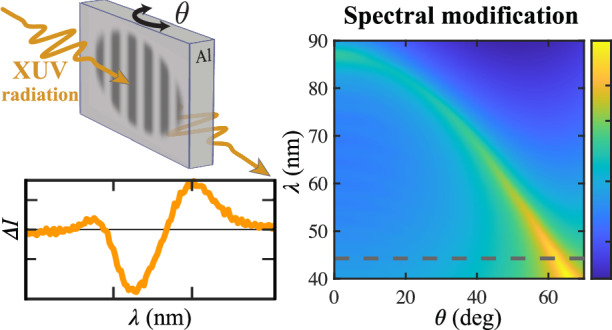

## Introduction

High peak-power pulsed laser sources are crucial for harnessing nonlinear (NL) effects, which enable a wide array of photonic applications, including harmonic generation^1^, self-action^2^, parametric amplification^3^, all-optical modulation^4^, and more. Historically, the availability of pulsed laser sources has constrained the highest pump photon frequencies for these applications to the near- and middle-ultraviolet region. The development of free electron lasers (FELs) and high harmonic generation (HHG) sources in the extreme ultraviolet (XUV) and X-ray spectral regions has opened the door to surpass these limitations^5–7^. These advancements are propelling the emergence of a radically new science named extreme NL optics (NLO), which is promising for radiation manipulation, attosecond metrology, and innovative spectroscopy techniques, e.g., NL spectroscopy, coherent multidimensional spectroscopy, soft X-ray photo-ionisation, and time-resolved XUV transient absorption^8–11^. These emerging ultrafast techniques enable the probing of molecules and condensed-phase materials by high-energy photons in the XUV and are unlocking new opportunities for our basic understanding of ultrafast relaxation, non-equilibrium processes, and chemical reactions^12^. However, the inherently weak NL light-matter interaction at such high photon energies, combined with the limited power of current XUV tabletop sources, hampers their disruptive potential for ultrafast spectroscopy and extreme NLO, which remain among the major challenges of photonics today.

At lower photon energies, plasmonics is fuelling advances in NLO by metal-based nanostructures capable of boosting light-matter interactions at the nanoscale^13^. Moreover, nanophotonics enables the engineering of the optical response by nano-structuring the dielectric properties of materials, which constitutes the basic ground for the development of metamaterials^14,15^ and metasurfaces^16^. These artificial “materials” embed nano-scaled structures arranged in periodic patterns. The shape and composition of the underpinning components can be engineered in order to achieve epsilon-near-zero (ENZ) regime^17^, otherwise accessible in natural ENZ materials displaying bulk phonon or plasmon resonances^18^. In terms of NL applications, ENZ materials offer extraordinary possibilities, owing to the tremendous field enhancement that they can provide^19–22^. Indeed, for low-absorbing media, exhibiting low dielectric permittivity modulus (vanishing for non-absorbing media), NL effects become non-perturbative, thus opening novel avenues for strong-field physical applications^19^. In contrast to surface plasmons, where field enhancement arises from localisation, ENZ thin films naturally enhance transverse-magnetic (TM) electric fields owing to the continuity of the normal component of the displacement vector ^23^. This process is resonant at a peculiar incidence angle where excitation of Ferrell-Berreman (FB) modes –surface-like excitations resembling bulk longitudinal plasmons^24^– is matched. In turn, such a resonant NL process can be grasped by an enhanced effective second-order NL coefficient^19,25^ accounting for FB mode excitation. From a physical standpoint, ENZ media support superluminal phase velocity and highly reduced group velocity, the so-called slow-light regime, and in turn enhance the NL light-matter interaction owing to the longer interaction time^23,26^. Currently, ENZ materials operate efficiently only in the near and mid infra-red (IR) frequency part of the spectrum, and, although potentially disruptive, their XUV functionality remains unexplored.

**Fig. 1:**
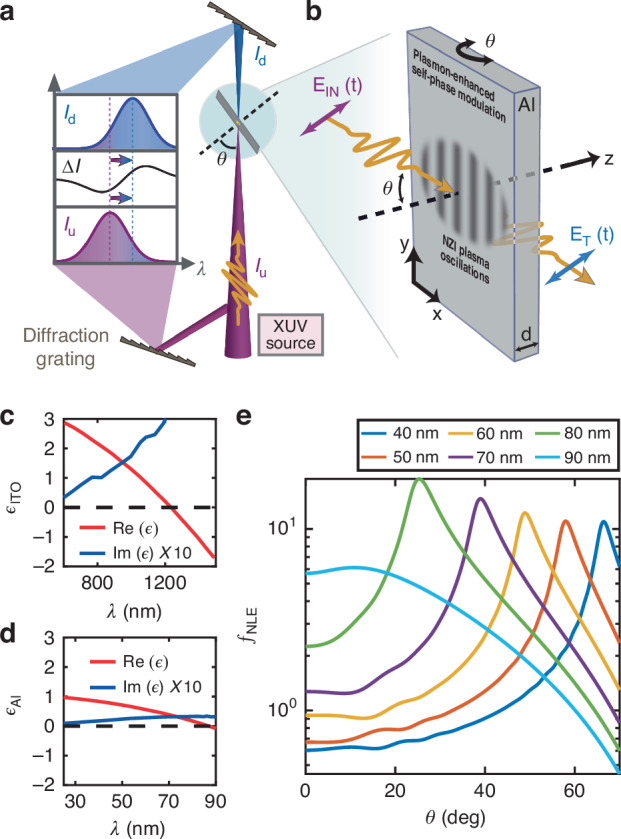
Experimental scheme and NZI NL enhancement. **a** Sketch of the FERMI EIS-TIMEX experimental setup enabling single shot spectral detection upstream (*I*_u_(*λ*)) and downstream (*I*_d_(*λ*)) the interaction of an XUV focused pulse with an Al free-standing thin foil of thickness *d* = 300 nm. The dependence of NL enhancement over the incidence angle *θ* is quantified by the difference of normalised spectra (Δ*I*(*λ*) = *I*_d_(*λ*) − *I*_u_(*λ*)). **b** Schematic illustration of FB modes produced by plasma oscillations in the NZI regime, enhancing nonlinearity analogously to ITO-based ENZ thin films^19^, working in the near infra-red regime, see (**c**). **c** The real and imaginary parts of *ϵ*_ITO_(*λ*). The ENZ condition is attained at ${\rm{Re}}[{\epsilon }_{{\rm{ITO}}}({\lambda }_{{\rm{ENZ}}}^{{\rm{ITO}}})]=0$. **d** *ϵ*_Al_(*λ*) displaying ENZ behaviour for *λ* ~85 nm. **e** Simulation of the *θ* dependence of the normalised NL enhancement factor *f*_NLE_ for different XUV carrier wavelengths *λ*, illustrating plasmon-enhanced SPM by *λ*-dependent FB resonance at *θ*_NZI_(*λ*)

Here we report the first evidence of ENZ-based NL enhancement in the XUV spectral regime. In particular, we demonstrate that self-driven spectral modulation^27^ is enhanced by broadband FB modes^23,24^ in aluminium (Al) thin foils, occurring at the near-zero effective-index (NZI) condition

1 $${\rm{Re}}\sqrt{{\epsilon }_{{\rm{Al}}}(\lambda )-{\sin }^{2}{\theta }_{{\rm{NZI}}}(\lambda )}=0$$

where *ϵ*_Al_(*λ*) is the relative dielectric permittivity of Al, reported in Fig. 1d. Specifically, by means of the FEL of the Trieste FERMI facility, we observe at the carrier wavelength *λ* ≃ 44 nm a strong self-phase modulation (SPM) enhancement dependent on incidence angle *θ*. For the investigated Al thin foils, the NZI FB-resonance *θ* = *θ*_NZI_(*λ*) arises over the entire 25 nm < *λ* < 85 nm broadband range as an effect of the mitigated Al absorption. Self-induced NL spectral modulation in Al mainly results from ultrafast heating that is modelled by an effective temporally nonlocal and nonlinearly saturated polarisation. Theoretical predictions based on such an effective model find experimental confirmation in the angle and power dependence of the transmitted spectrum.

## Results and discussion

The FERMI EIS-TIMEX experimental setup, realised to measure plasmon-enhanced SPM by FB mode excitation, is sketched in Fig. 1a, b. The experimental approach employs the interaction of TM polarised FEL pulses (by a single-shot raster scan reported in Materials and Methods) with a pristine Al free-standing foil of thickness *d* = 300 nm, at an incidence angle *θ*. The FEL pulse associated electric field can be expressed as ${{\bf{E}}}_{{\rm{IN}}}({\bf{r}},t)={\rm{Re}}[{{\bf{E}}}_{0}({\bf{r}},t){e}^{i{{\bf{k}}}_{{\bf{0}}}\cdot {\bf{r}}-i{\omega }_{0}t}]$, where the vectorial envelope **E**_0_(**r**, *t*) lies in the *x-z* plane spanned by the ${\hat{{\bf{e}}}}_{x,z}$ unit vectors, ${{\bf{k}}}_{0}=(2\pi /\lambda ){\hat{{\bf{k}}}}_{0}$ is the carrier wavevector with unit vector ${\hat{{\bf{k}}}}_{0}=\cos \theta {\hat{{\bf{e}}}}_{z}+\sin \theta {\hat{{\bf{e}}}}_{x}$, *λ* and *ω*_0_ are the carrier wavelength and angular frequency, respectively. Owing to the large FEL peak intensity ${I}_{{\rm{peak}}}=(1/2){\epsilon }_{0}c| {{\bf{E}}}_{0}({\bf{r}},t){| }_{\max }^{2} > 200$ TW/cm^2^ (where *ϵ*_0_ is the vacuum dielectric permittivity and *c* is the speed of light in vacuum), electrons in Al undergo ultrafast heating followed by relaxation via electron-phonon scattering^28^. Such complex electron dynamics are characterised by a photo-induced transient electronic temperature *T*_e_(**r**, *t*), leading, in turn, to a spatio-temporal refractive index modulation. We phenomenologically account for ultrafast electron heating by a NL polarisation ${{\bf{P}}}_{{\rm{NL}}}({\bf{r}},t)={\epsilon }_{0}{\rm{Re}}\left[\Delta {\epsilon }_{{\rm{NL}}}({\bf{r}},t)A({\bf{r}},t)\hat{{\bf{n}}}{e}^{i{k}_{0}(x\sin \theta +\tilde{n}z-ct)}\right]$, where $\tilde{n}(\lambda ,\theta )=\sqrt{{\epsilon }_{{\rm{Al}}}(\lambda )-{\sin }^{2}\theta }$ is the effective refractive index, *A*(**r**, *t*) is the vectorial electric field envelope within the Al foil, and $\hat{{\bf{n}}}$ is the polarisation unit vector. Finally, Δ*ϵ*_NL_(**r**, *t*) accounts for the NL dielectric permittivity modulation, which originates from coherent electron dynamics (providing instantaneous Kerr effect), the delayed thermal nonlinearity produced by ultrafast heating, and the saturation due to electron-phonon collision quenching^29^, see Materials and Methods for further details. The latter saturation effect, not observed in ref. ^27^, is unveiled by the enhancement induced by NZI resonance. The NL dielectric permittivity correction Δ*ϵ*_NL_(**r**, *t*) produces asymmetric redshifted spectral broadening due to the combination of the above mentioned effects, analogously to stimulated Raman-shifted SPM in dielectrics^30^. In our measurements, the NL-induced spectral modulation of the transmitted pulses is quantified by the difference of normalised spectra upstream (*I*_u_(*λ*)) and downstream (*I*_d_(*λ*)) of the sample (Δ*I*(*λ*) = *I*_d_(*λ*) − *I*_u_(*λ*))^27,31^, see Materials and Methods. Owing to the ENZ permittivity *ϵ*_Al_(*λ*) of Al around the plasma frequency (corresponding to *λ*_P_ ~85 nm)^32–35^, the FEL pulses excite FB modes enabling transient radiation trapping^23,24^ and NL enhancement^19^. In turn, while in Indium tin oxide (ITO) FB mode excitation mainly occurs at the plasma frequency (arising at the ENZ condition reported in Fig. 1c and is angle insensitive)^24^, in Al it occurs over the entire ENZ region (where ${\rm{Re}}\left[{\epsilon }_{{\rm{Al}}}(\lambda )\right] < 1$, i.e. 25 nm < *λ* < *λ*_P_) for distinct excitation angles *θ*_NZI_(*λ*) matching the NZI condition in Eq. (1), benefiting from the reduced absorption of Al, accounted for by ${\rm{Im}}[{\epsilon }_{{\rm{Al}}}(\lambda )]$, reported in Fig. 1d. Such a broadband resonant FB mode excitation in the Al-based foil produces a NL enhancement *f*_NLE_ ≃ 20, see Fig. 1e and Materials and Methods. Such a parameter accounts for the boosting of electric field intensity within the ENZ material and its reduced effective refractive index, see Materials and Methods, thus quantifying the enhancement of third-order NL effects. As illustrated in Fig. 1e, for every fixed wavelength *λ* < *λ*_P_, *f*_NLE_ is maximised at a peculiar angle *θ*_NZI_(*λ*) such that Eq. (1) is satisfied.

**Fig. 2:**
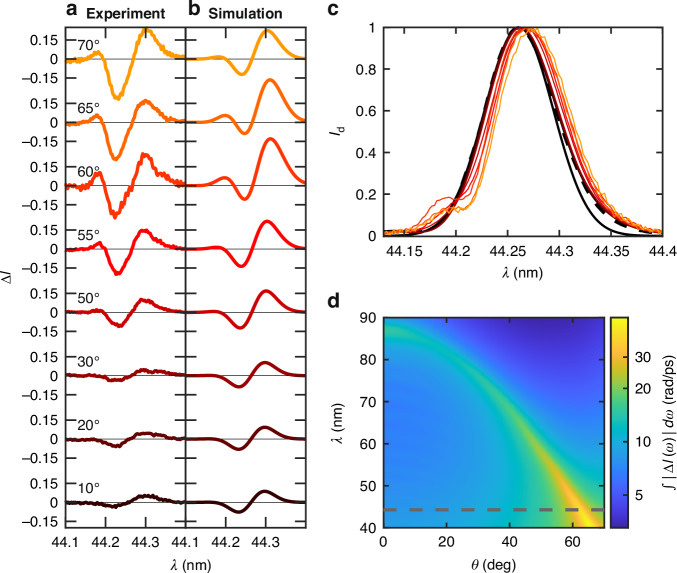
NZI enhancement of spectral modulation by impinging angle tuning. **a** Experimental and **b** theoretical variation Δ*I*(*λ*) of normalised spectra for several distinct impinging angles *θ*, with pulse peak intensities and duration fixed to 267 TW/cm^2^ and 37 fs, respectively. **c** Averaged normalised downstream (transmitted) spectra *I*_d_(*λ*) for several distinct impinging angles *θ* (the colour code coincides with **a**, **b**). The impinging experimental and theoretical spectra are indicated with black dashed and full lines, respectively. The experimental spectrum is measured without the sample while the theoretical one, used for the simulation, is a transform-limited 37 fs Gaussian pulse. **d** Dependence of the theoretically predicted CSM, quantified by $\mathop{\int}\nolimits_{0}^{\infty }| \Delta I(\omega )| d\omega$, over the incidence angle *θ* and the carrier wavelength *λ* for fixed peak intensity 267 TW/cm^2^ and 37 fs pulse duration. The black dashed horizontal line indicates the wavelength adopted in the experimental measurement reported in (**a**)

The experimental evidence of ENZ-based NL enhancement of self-driven spectral modulation is summarised in Fig. 2a, illustrating the measured normalised spectral difference, Δ*I*(*λ*), for diverse excitation angles and fixed FEL pulse fluence of 10.5 J/cm^2^ and time duration of 37 fs full width at half maximum (FWHM), corresponding to a peak intensity of 267 TW/cm^2^. In agreement with the expected enhancement depicted in Fig. 1e, the spectral modulation signal at *θ* = 60^∘^ is amplified by the FB mode excitation. As an effect of the delayed thermal nonlinearity of out-of-equilibrium hot conduction electrons in Al^29^, the spectral modulation is dominated by a strong redshift, also evident in the transmitted spectra reported by coloured lines in Fig. 2c. Moreover, the shoulder-like feature across 44.18 nm highlights a resonant spectral broadening around *θ* = 60^∘^, ascribable to instantaneous SPM (ISPM)^36^. Our theoretical model accounts for both of these mechanisms through the effective NL polarisation **P**_NL_(**r**, *t*), similarly to previously reported NL spectral broadening in Mg below the absorption edge of core electrons^27^. Figure 2c also illustrates the comparison between theoretically assumed and experimentally observed upstream spectra in solid and dashed black lines, respectively, highlighting excellent agreement. The theoretically predicted spectral modulation is depicted in Fig. 2b for the same incidence angles adopted in the experiments reported in Fig. 2a. In particular, experimental data are reproduced by a thermal ratio *f*_Th_ = 0.996, i.e., the relative weight of delayed thermal response and saturated instantaneous Kerr nonlinearity. However, for the considered FEL pulse peak intensity, the temperature saturation significantly attenuates the delayed nonlinearity contribution (see Fig. 4 for further details). Figure 2d further illustrates the *λ*, *θ* dependence of the cumulative spectral modulation (CSM), quantified by $\mathop{\int}\nolimits_{0}^{\infty }| \Delta I(\omega )| d\omega$. Similarly to Fig. 1e, the CSM enhancement is maximised at *θ*_NZI_(*λ*) such that Eq. (1) is satisfied. Notably, the NL polarisation saturation induces a blurring of *θ*_NZI_(*λ*), especially for short wavelengths. Interestingly, the intensity of the signal is larger for shorter wavelengths, owing to reduced absorption accounted for by a lower ${\rm{Im}}[{\epsilon }_{{\rm{Al}}}(\lambda )]$ in such frequency region (see Fig. 1d).

**Fig. 3:**
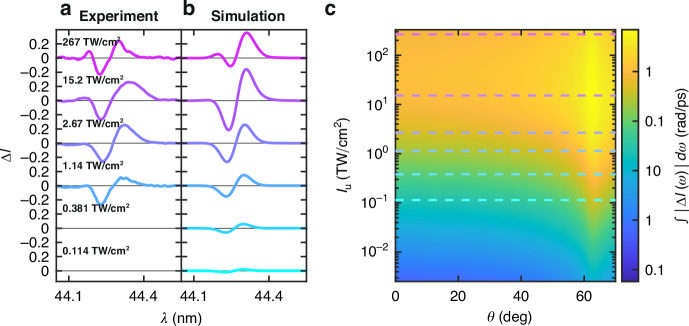
Experimental/theoretical NL signal power dependence. Comparison between the **a** experimental and **b** theoretical modulation of normalised spectral intensity (Δ*I*(*λ*)) at *θ* = 60^∘^. The experimental results are reported above 1.14 TW/cm^2^, which corresponds to the lowest peak intensity capable of producing a detectable signal by the downstream spectrometer. **c** Dependence of the theoretically predicted CSM over the incidence angle *θ* and the FEL peak intensity for carrier wavelength *λ* = 44.26 nm and 37 fs pulse duration

Besides spectral blurring, NL polarisation saturation strongly affects the FEL peak intensity dependence of SPM. As illustrated in Fig. 3a, the SPM signal increases with the FEL peak intensity in a saturated fashion even at the lowest considered FEL intensity, i.e., the one producing the minimum signal detectable by the downstream spectrometer (1.14 TW/cm^2^). However, a drastic ISPM suppression is observed upon FEL peak intensity reduction, quantified by the peak around 44.18 nm (see Fig. 3a). This saturated dependence of the NL signal is reproduced by our phenomenological model obtained with the same parameters as in Fig. 2b. The excellent agreement with the experimental results demonstrates the accuracy and predictive power of spectral modulation models (see Fig. 3b). Moreover, the theoretical model demonstrates the possibility of observing SPM signal in the NZI condition even at 380 GW/cm^2^. The dependence of the CSM intensity on the incidence angle and the peak intensity of the FEL pulse is illustrated in Fig. 3c. To rationalise this agreement, we should consider that the NL redshift is related to the material optical properties modulation over the FEL pulse timescale, producing instantaneous and delayed temperature-dependent saturation. In turn, the time-dependent NL saturation occurring during pulse propagation produces a NL signal mainly from the leading part of the pulse. The strong saturation of delayed thermal nonlinearity achieved at the peak intensity of 267 TW/cm^2^ implies a smaller NL modulation than that produced at 15 TW/cm^2^. Differently, for ISPM, the presence of only one saturation mechanism implies an increase in the NL signal with the peak intensity. Such distinct saturation mechanisms, observed for delayed thermal and instantaneous Kerr nonlinearities, can also help to rationalise previously reported results concerning SPM below the absorption edge of metallic Mg^27^.

The reported NZI nonlinearity enhancement at remarkably low FEL intensities opens the door to new investigations of NL phenomena with tabletop HHG-based systems, e.g., by means of experimental schemes similar to the one illustrated in Fig. 1a. Specifically, this could be achieved by focusing HHG radiation on a tilted thin film of Al, producing sub-nm spectral redshifts analogously to the results reported here. Since the FB resonant excitation angle *θ*_NZI_(*λ*) strongly depends on *λ* (see Fig. 2d), each harmonic of the HHG system would then experience a different shift arising from the NL enhancement of the effect. The capability to obtain from a tabletop XUV system two beams with tuneable sub-nm frequency shift can overcome current limitations in frequency resolution dictated by the IR pump generating the HHG spectrum^37^, thus enabling the realisation of innovative pump-probe schemes with unprecedented resolution at specific electronic resonances.

Moreover, HHG occurring in reflection via the plasma mirror effect^38^ can, in principle, be enhanced by FB excitation in the Al thin foils reported here, thus providing further potential applications in the advancement of XUV strong-field physics. However, spectral measurements in reflection from a rotating sample are currently beyond the capabilities of the FEL Fermi facility and will require further advancements in order to investigate HHG via plasma mirror.

Interestingly, although Al is a centrosymmetric material, second-order nonlinearities may still arise due to symmetry breaking induced by photon momentum at the surface under tilted excitation. In addition, engineered non-centrosymmetric Al samples –achieved, for instance, through strain engineering, surface patterning, or by coating with non-centrosymmetric materials such as LiNbO_3_^10^ –could be employed to enable NZI-enhanced second-order NL processes. This possibility could offer qualitatively new insights into NZI-XUV NLO, particularly due to the ability of ENZ media to relax phase-matching constraints, and thus merits further investigation.

## Conclusions

In summary, we have presented the first experimental evidence of ENZ-based NL enhancement in the XUV spectral range. Our work demonstrates that by leveraging the unique properties of ENZ materials, specifically through the excitation of FB modes, it is possible to achieve amplified NL effects such as SPM at remarkably low FEL peak intensities, as low as 380 GW/cm^2^. By carefully tilting the sample to control the excitation of these modes, we achieved resonant enhancement of SPM, which opens up new opportunities for manipulating light-matter interactions in the XUV domain.

The observed SPM effects arise from a combination of two distinct processes: an ISPM that results in a spectral shoulder at 44.18 nm, and a delayed thermal response, which induces a spectral redshift. This dual mechanism underscores the complex dynamics achievable in ENZ materials, where both instantaneous and time-dependent nonlinearities contribute to spectral modulation. Our findings thus provide a novel approach for controlling and enhancing inherently weak nonlinearities in the XUV spectral range, which has historically presented challenges due to the typical submicrometric penetration depth and the low NL response of materials in such a spectral domain^8^.

The NZI enhancement offers an innovative nanophotonic platform for achieving efficient SPM in this extreme spectral regime, unlocking new potential for strong-field physics applications using XUV photons, such as ultrafast spectroscopic techniques and HHG. Furthermore, we anticipate that XUV plasmon enhancement could enable efficient harmonic generation, wave mixing, and transient grating formation, and offer a new approach for advancing XUV NL nanospectroscopy in tabletop setups with relatively low peak power, such as HHG sources. This development could ultimately broaden access to XUV NL optical techniques, enabling advanced studies of matter under extreme conditions and pushing the frontier of nanoscale spectroscopy and ultrafast photonics.

## Materials and Methods

### Experimental setup and data treatment

As described in ref. ^27^, the experimental setup is based on nearly transform-limited XUV pulses provided by the FERMI FEL facility of Trieste. The emitted pulses have a wavelength of ~44.26 nm with TM linear polarisation, a maximum fluence of 10.5 J/cm^2^, a pulse duration FWHM of 37 fs, and a repetition rate of ~1 Hz. The attenuation of the FEL beam was performed by inserting free-standing metallic foils into the beam path. The experimental setup consists of two XUV single-shot spectrometers positioned upstream (PRESTO) and downstream (WEST)^31^ of an experimental chamber where the sample is housed. The FEL beam is focused on the sample by a gold-coated ellipsoidal mirror with a focal length of 1400 mm. This setup achieves tight focus, producing a spot with a diameter of approximately ~11 μm. Owing to the permanent damage induced by the Al absorption of intense FEL pulses, the measurements were carried out in a single-shot raster mode. After each FEL pulse, selected by a mechanical shutter, the sample is moved to an unexposed region using an automated 5-axis manipulator, ensuring both the focus and the normal incidence of the FEL beam are preserved. Normalised spectra before (*I*_u_(*λ*)) and after (*I*_d_(*λ*)) the sample were collected for each single FEL shot. Due to the inherent instability of the FEL central wavelength (~0.02 nm), *I*_u_(*λ*) and *I*_d_(*λ*) were equally shifted in wavelength to maximise the spectral overlap of different shots with respect to a specific reference *I*_u_(*λ*) spectrum. After a subsequent normalisation to the maximum intensity, a direct difference of normalised spectra was performed, Δ*I*(*λ*) = *I*_d_(*λ*) − *I*_u_(*λ*). The measured spectra, reported in Figs. 2 and 3, are obtained by averaging single-shot spectral differences Δ*I*(*λ*) on ~70 FEL shots.

### Calculation of electric field in the foil

In this section, we report the details of the calculation of the electric field in the Al slab, which plays a fundamental role in the evaluation of the nonlinearity enhancement factor. The nanometric Al slab is considered to be oriented perpendicular to the *z-*axis, located between *z* = 0 and *z* = *d*. The FEL pulse is TM polarised, implying that the impinging electric field ${{\bf{E}}}_{{\rm{IN}}}({\bf{r}},t)={\rm{Re}}\left[{E}_{0}\left(\cos \theta {\hat{{\bf{e}}}}_{x}-\sin \theta {\hat{{\bf{e}}}}_{z}\right){e}^{i{k}_{0}(x\sin \theta +z\cos \theta )-i{\omega }_{0}t}\right]$ lies in the *x* − *z* plane (see Fig. 1b), where *θ* is the incidence angle, ${\hat{{\bf{e}}}}_{x,z}$ are the *x*, *z* unit vectors, *ω*_0_ and *k*_0_ = *ω*_0_/*c* are the carrier angular frequency and wavenumber, respectively, and *c* is the speed of light in vacuum. The electromagnetic field in all space can be expressed as

2 $${\bf{E}}\left({\bf{r}},t\right)=\left\{\begin{array}{ll}{\rm{Re}}\left[\begin{array}{l}{E}_{0}\left(\cos \theta {\hat{{\bf{e}}}}_{x}-\sin \theta {\hat{{\bf{e}}}}_{z}\right){e}^{i{k}_{0}\left(x\sin \theta +z\cos \theta \right)-i{\omega }_{0}t}+\\ +{E}_{{\rm{R}}}\left(\cos \theta {\hat{{\bf{e}}}}_{x}+\sin \theta {\hat{{\bf{e}}}}_{z}\right){e}^{i{k}_{0}\left(x\sin \theta -z\cos \theta \right)-i{\omega }_{0}t}\end{array}\right]\quad &z < 0\\ {\rm{Re}}\left[\begin{array}{l}\left({E}_{+x}{\hat{{\bf{e}}}}_{x}-\frac{\sin \theta }{\tilde{n}}{E}_{+x}{\hat{{\bf{e}}}}_{z}\right){e}^{i{k}_{0}(x\sin \theta +\tilde{n}z)-i{\omega }_{0}t}+\\ +\left({E}_{-x}{\hat{{\bf{e}}}}_{x}+\frac{\sin \theta }{\tilde{n}}{E}_{-x}{\hat{{\bf{e}}}}_{z}\right){e}^{i{k}_{0}(x\sin \theta -\tilde{n}z)-i{\omega }_{0}t}\end{array}\right]\quad &0 < z < d\\ {\rm{Re}}\left[{E}_{{\rm{T}}}\left(\cos \theta {\hat{{\bf{e}}}}_{x}-\sin \theta {\hat{{\bf{e}}}}_{z}\right){e}^{i{k}_{0}\left(x\sin \theta +z\cos \theta \right)-i{\omega }_{0}t}\right]\quad &z > d\end{array}\right.$$

3 $${\bf{B}}\left({\bf{r}},t\right)=\left\{\begin{array}{ll}\frac{1}{c}{\rm{Re}}\left[\left({E}_{0}{e}^{i{k}_{0}z\cos \theta }-{E}_{{\rm{R}}}{e}^{-i{k}_{0}z\cos \theta }\right){\hat{{\bf{e}}}}_{y}{e}^{i{k}_{0}x\sin \theta -i{\omega }_{0}t}\right]\quad &z < 0\\ \frac{1}{c}{\rm{Re}}\left[\frac{{\epsilon }_{{\rm{Al}}}}{\tilde{n}}\left(\begin{array}{l}{E}_{+x}{e}^{i{k}_{0}\tilde{n}z}-{E}_{-x}{e}^{-i{k}_{0}\tilde{n}z}\end{array}\right){\hat{{\bf{e}}}}_{y}{e}^{i{k}_{0}x\sin \theta -i{\omega }_{0}t}\right]\quad &0 < z < d\\ \frac{1}{c}{\rm{Re}}\left[{E}_{{\rm{T}}}{e}^{i{k}_{0}(x\sin \theta +z\cos \theta )-i{\omega }_{0}t}{\hat{{\bf{e}}}}_{y}\right]\quad &z > d\end{array}\right.$$

where *ϵ*_Al_(*λ*) is the relative dielectric permittivity of Al, depicted in Fig. 1d, dependent on the vacuum wavelength *λ* = 2*πc*/*ω*_0_, and $\tilde{n}(\lambda )=\sqrt{{\epsilon }_{{\rm{Al}}}(\lambda )-{\sin }^{2}\theta }$ is the effective refractive index within the Al slab. Applying the boundary conditions for the continuity of (i) the *x* and *y* components of electric and magnetic fields, and (ii) the *z* component of the displacement vector, one gets

4 $$\begin{array}{ll}{E}_{+x}=\frac{2\tilde{n}\cos \theta \left({\epsilon }_{{\rm{Al}}}\cos \theta +\tilde{n}\right){e}^{-2i{k}_{0}\tilde{n}d}}{{\left({\epsilon }_{{\rm{Al}}}\cos \theta +\tilde{n}\right)}^{2}{e}^{-2i{k}_{0}\tilde{n}d}-{\left(\tilde{n}-{\epsilon }_{{\rm{Al}}}\cos \theta \right)}^{2}}{E}_{0}\\ {E}_{-x}=\frac{2\tilde{n}\left({\epsilon }_{{\rm{Al}}}\cos \theta -\tilde{n}\right)\cos \theta }{-{\left(\tilde{n}-{\epsilon }_{{\rm{Al}}}\cos \theta \right)}^{2}+{\left({\epsilon }_{{\rm{Al}}}\cos \theta +\tilde{n}\right)}^{2}{e}^{-2i{k}_{0}\tilde{n}d}}{E}_{0}\\ {E}_{+z}=-\frac{\sin \theta }{\tilde{n}}\frac{2\tilde{n}\cos \theta \left({\epsilon }_{{\rm{Al}}}\cos \theta +\tilde{n}\right){e}^{-2i{k}_{0}\tilde{n}d}}{{\left({\epsilon }_{{\rm{Al}}}\cos \theta +\tilde{n}\right)}^{2}{e}^{-2i{k}_{0}\tilde{n}d}-{\left(\tilde{n}-{\epsilon }_{{\rm{Al}}}\cos \theta \right)}^{2}}{E}_{0}\\ {E}_{-z}=\frac{\sin \theta }{\tilde{n}}\frac{2\tilde{n}\left({\epsilon }_{{\rm{Al}}}\cos \theta -\tilde{n}\right)\cos \theta }{-{\left(\tilde{n}-{\epsilon }_{{\rm{Al}}}\cos \theta \right)}^{2}+{\left({\epsilon }_{{\rm{Al}}}\cos \theta +\tilde{n}\right)}^{2}{e}^{-2i{k}_{0}\tilde{n}d}}{E}_{0}\end{array}$$

The calculated field at the entrance of the Al slab is then given by

5 $${{\bf{E}}}_{{\rm{Al}}}(x,z={0}^{+},t)={\rm{Re}}\left[{f}_{{\rm{E}}}{E}_{0}{e}^{i{k}_{0}x\sin \theta -i{\omega }_{0}t}\hat{{\bf{n}}}\right]$$

where

6 $$\hat{{\bf{n}}}=\frac{({E}_{+x}+{E}_{-x}){\hat{{\bf{e}}}}_{x}+(\sin \theta /\tilde{n})({E}_{-x}-{E}_{+x}){\hat{{\bf{e}}}}_{z}}{\sqrt{{\left\vert {E}_{+x}+{E}_{-x}\right\vert }^{2}+({\sin }^{2}\theta /\left\vert {\tilde{n}}^{2}\right\vert ){\left\vert {E}_{-x}-{E}_{+x}\right\vert }^{2}}}$$

and

7 $${f}_{{\rm{E}}}=\frac{\sqrt{{\left\vert {E}_{+x}+{E}_{-x}\right\vert }^{2}+\frac{{\sin }^{2}\theta }{\left\vert {\tilde{n}}^{2}\right\vert }{\left\vert {E}_{-x}-{E}_{+x}\right\vert }^{2}}}{{E}_{0}}$$

We account for pulse propagation by the slowly varying envelope approximation (SVEA), where the electric field within the Al slab input interface

8 $${{\bf{E}}}_{{\rm{Al}}}(x,z=0,t)={\rm{Re}}\left[A(x,z=0,t){e}^{i{k}_{0}(x\sin \theta -ct)}\hat{{\bf{n}}}\right]$$

where

9 $$A(x,z=0,t)={f}_{{\rm{E}}}{E}_{0}(x,z=0,t)$$

is the envelope. The Fourier transform condition of FEL pulses implies an impinging envelope $A(x,z=0,t)={f}_{E}{E}_{0}(z=0,t){e}^{-{(t-x\sin \theta /c)}^{2}/(2{{t}_{w}}^{2})}$, where *t*_*w*_ = 22 fs is the electric field time duration arising from a FEL intensity profile of duration 37 fs (full width half maximum). Indeed, owing to the pulsed nature of the XUV radiation impinging the Al slab, the pulse is localised around a time-dependent spatial spot $\Delta x > \Delta {x}_{\min }$ with minimum dimension $\Delta {x}_{\min }\simeq c{t}_{w}\simeq 7\,\mu {\rm{m}} > >$ 50 nm, which is much larger than the XUV pulse carrier wavelength. In turn, we can safely neglect the spatial shift of the envelope induced by tilted propagation and evaluate its NL evolution parametrically for every *x*.

### Calculation of the NL response

The effect of nonlinearity is accounted for by the NL polarisation

10 $$\begin{array}{l}{{\bf{P}}}_{{\rm{NL}}}({\bf{r}},t)=\\{\epsilon }_{0}{\rm{Re}}\left[\Delta {\epsilon }_{{\rm{NL}}}[| A({\bf{r}},t){| }^{2}]A({\bf{r}},t)\hat{{\bf{n}}}{e}^{i{k}_{0}(x\sin \theta +\tilde{n}z-ct)}\right]\end{array}$$

where the NL dielectric permittivity correction Δ*ϵ*_NL_[∣*A*(**r**, *t*)∣^2^] takes into account the instantaneous Kerr effect ensuing from coherent electron dynamics, delayed thermal nonlinearity produced by ultrafast heating^27^, and saturation due to electron-phonon collision quenching^29^. Explicitly, it can be expressed as Δ*ϵ*_NL_[∣*A*(**r**, *t*)∣^2^] = Δ*ϵ*_Kerr_[∣*A*(**r**, *t*)∣^2^] + Δ*ϵ*_Th_[∣*A*(**r**, *t*)∣^2^], where $\Delta {\epsilon }_{{\rm{Kerr}}}[| A({\bf{r}},t){| }^{2}]={\chi }^{(3)}(1-{f}_{{\rm{Th}}})| A({\bf{r}},t){| }^{2}/[1+| A({\bf{r}},t){| }^{2}/{A}_{{\rm{S}}}^{2}]$ and $\Delta {\epsilon }_{{\rm{Th}}}[| A({\bf{r}},t){| }^{2}]={f}_{{\rm{Th}}}{\chi }^{(3)}{I}_{{\rm{Th}}}({\bf{r}},t)/[1+{I}_{{\rm{Th}}}({\bf{r}},t)/{A}_{{\rm{Th}}}^{2}]$.

Specifically, ${I}_{{\rm{Th}}}({\bf{r}},t)=\mathop{\int}\limits^{\infty }d{t}^{{\prime} }{h}_{{\rm{Th}}}\left({t}^{{\prime} }\right){\left\vert A\left({\bf{r}},t-{t}^{{\prime} }\right)\right\vert }^{2}/[1+| A({\bf{r}},t){| }^{2}/{A}_{{\rm{S}}}^{2}]$, ${h}_{{\rm{Th}}}\left(t\right)={\left({\tau }_{{\rm{Th}}}-{\tau }_{{\rm{r}}}\right)}^{-1}\left({e}^{-t/{\tau }_{{\rm{Th}}}}-{e}^{-t/{\tau }_{{\rm{r}}}}\right)$ is the thermal response function^27^, *χ*^(3)^ is the third-order susceptibility, *f*_Th_ is the thermal nonlinearity fraction with respect to ISPM, and *A*_S_, *A*_Th_ are characteristic fields accounting for the saturation of the instantaneous Kerr effect (*A*_S_) and of the delayed nonlinearity (*A*_Th_). We evaluate radiation propagation within the Al foil by deriving a Generalised Nonlinear Schrödinger Equation (GNLSE) for the envelope *A*(**r**, *t*). Starting from Maxwell’s equations accounting for **P**_NL_(**r**, *t*), radiation evolution is described by the inhomogeneous double-curl equation ∇ × ∇ × **E** = − *μ*_0_∂^2^**P**_NL_/∂*t*^2^ − [*ϵ*_Al_(*ω*_0_)/*c*^2^]∂^2^**E**/∂*t*^2^. Thus, in the SVEA, we obtain

11 $${\partial }_{z}A\left({\bf{r}},t\right)=i\frac{{\omega}_{0}}{2c\left\vert \tilde{n}\right\vert }{e}^{-2\frac{{\omega }_{0}}{c}\,{\rm{Im}}\left(\tilde{n}\right)z}\Delta {\epsilon }_{{\rm{NL}}}[| A({\bf{r}},t){| }^{2}]A\left({\bf{r}},t\right)$$

Such a GNLSE, governing the evolution of the envelope defined in Eq. (9), easily allows us to recognise the NL enhancement factor in the unsaturated limit ${f}_{{\rm{NLE}}}(\lambda ,\theta )={\left\vert {f}_{{\rm{E}}}(\lambda ,\theta )\right\vert }^{2}/\left\vert \tilde{n}(\lambda ,\theta )\right\vert$. The angle and wavelength dependencies of *f*_NLE_(*λ*, *θ*) are reported in Fig. 1. The results reported are attained by the numerical solution of Eq. (11) by a fourth-order Runge-Kutta algorithm. To reproduce the experimental data in Figs. 2 and 3, we adopted the parameters

- *χ*^(3)^ = 4.5 ⋅ 10^−21^ m^2^/V^2^,
- *f*_Th_ = 0.9965,
- *A*_S_ = 2.6 ⋅ 10^10^ V/m,
- *A*_Th_ = 2.2 ⋅ 10^9^ V/m.

**Fig. 4:**
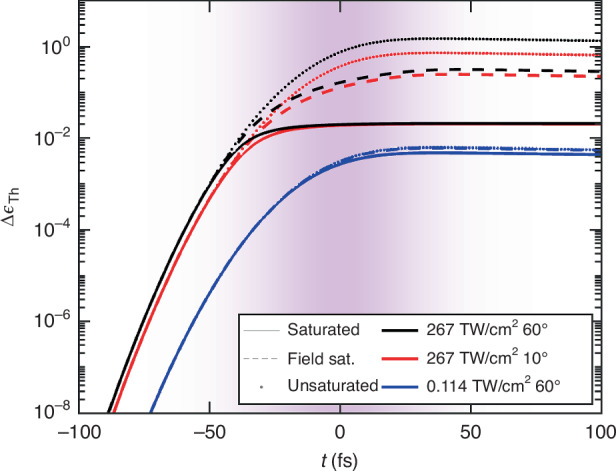
Time evolution of **Δ*ϵ***_Th_ for different saturation conditions. The dotted, dashed and solid lines represent Δ*ϵ*_Th_ without saturation effects, with field saturation only, and with both saturation mechanisms, respectively. The time profiles associated with peak intensity of 267 TW/cm^2^ and incidence angles of 10^∘^ and 60^∘^ are reported by red and black lines, respectively. Finally, the time profile associated with peak intensity of 0.114 TW/cm^2^ and incidence angle of 60^∘^ is reported by blue lines. The purple area represents the intensity profile of the FEL beam, whose phase is modulated by NL effects

The retrieved thermal fraction *f*_Th_ indicates a dominant contribution of delayed thermal nonlinearity, strongly suppressed by the temperature saturation field accounted for by *A*_Th_. Figure 4 shows the temporal profile of the NL dielectric relative permittivity correction Δ*ϵ*_Th_[∣*A*(**r**, *t*)∣^2^] for different conditions of impinging peak intensity and angle *θ*. By comparing the red and black dotted lines, a large difference is observed in the pulse time window, testifying the large dependence of the NL effect on the angle. This difference decreases gradually owing to the two saturation processes. Specifically, over distinct excitation angles (*θ* = 10^∘^, and 60^∘^, indicated by red and black lines, respectively), Δ*ϵ*_Th_[∣*A*(**r**, *t*)∣^2^] is modified only by the leading part of the pulse. Differently, for low peak intensity (blue lines), Δ*ϵ*_Th_[∣*A*(**r**, *t*)∣^2^] modulation is less pronounced. However, the negligible role of saturation in such conditions produces a large modulation in the time window of maximum pulse intensity. For this reason, the FEL pulse intensity does not largely affect the spectral redshift.

## Data Availability

The data that support the findings of this study are available from the corresponding authors upon reasonable request.
